# Multi-focal HIFU reduces cavitation in mild-hyperthermia

**DOI:** 10.1186/s40349-017-0089-8

**Published:** 2017-04-13

**Authors:** Vandiver Chaplin, Charles F. Caskey

**Affiliations:** 1 Vanderbilt University Institute of Imaging Science, 1161 21st Avenue South, Nashville, TN 37232 USA; 2 Department of Radiology and Radiological Sciences, Vanderbilt University Medical Center, 1161 21st Avenue South, Nashville, TN 37232 USA

**Keywords:** High-intensity focused ultrasound (HIFU), Cavitation, Passive acoustic mapping, Hyperthermia

## Abstract

**Background:**

Mild-hyperthermia therapy (40–45 °C) with high-intensity focused ultrasound (HIFU) is a technique being considered in a number of different treatments such as thermally activated drug delivery, immune-stimulation, and as a chemotherapy adjuvant. Mechanical damage and loss of cell viability associated with HIFU-induced acoustic cavitation may pose a risk during these treatments or may hinder their success. Here we present a method that achieves mild heating and reduces cavitation by using a multi-focused HIFU beam. We quantify cavitation level and temperature rise in multi-focal sonications and compare it to single-focus sonications at the transducer geometric focus.

**Methods:**

Continuous wave sonications were performed with the Sonalleve V2 transducer in gel phantoms and pork at 5, 10, 20, 40, 60, 80 acoustic watts for 30 s. Cavitation activity was measured with two ultrasound (US) imaging probes, both by computing the raw channel variance and using passive acoustic mapping (PAM). Temperature rise was measured with MR thermometry at 3 T. Cavitation and heating were compared for single- and multi-focal sonication geometries. Multi-focal sonications used four points equally spaced on a ring of either 4 mm or 8 mm diameter. Single-focus sonications were not steered.

**Results:**

Multi-focal sonication generated distinct foci that were visible in MRI thermal maps in both phantoms and pork, and visible in PAM images in phantoms only. Cavitation activity (measured by channel variance) and mean PAM image value were highly correlated (r > 0.9). In phantoms, cavitation exponentially decreased over the 30-second sonication, consistent with depletion of cavitation nuclei. In pork, sporadic spikes signaling cavitation were observed with single focusing only. In both materials, the widest beam reduced average and peak cavitation level by a factor of two or more at each power tested when compared to a single focus. The widest beam reduced peak temperature by at least 10 °C at powers above 5 W, and created heating that was more spatially diffuse than single focus, resulting in more voxels in the mild heating (3–8 °C) range.

**Conclusions:**

Multi-focal HIFU can be used to achieve mild temperature elevation and reduce cavitation activity.

## Background

High intensity focused ultrasound (HIFU) is a technology with growing potential to provide palliative or curative treatment of tumorous diseases. Therapeutic HIFU is often classified by two primary modalities of cellular destruction: mechanical and thermal. These categories serve as broad conceptual guides to predict ultrasound-induced biological effects, based on the amplitude and degree of non-linearity of input acoustic fields. Different biophysical interactions may be preferred depending on the kind of HIFU treatment being applied.

Acoustic cavitation is the formation and subsequent collapse of small bubbles from gas nuclei in fluids due to high negative pressures. It occurs with both thermal and non-thermal HIFU and has been used to enhance thermal therapy by increasing the rate of temperature gain in the target medium. Cavitation is thought to accelerate heating through dissipation of the primary acoustic wave via viscoelastic and acoustic effects creating viscous shear flows at bubble-tissue interfaces, and generating energy at higher harmonics of the transmitted frequency which are preferentially absorbed in most tissues [1, 2]. Due to depletion of nucleation sites, movement of cavitation bubbles under radiation force, and inertial collapse at large rarefaction pressure, cavitation is a stochastic, transient phenomenon. The probability of forming an endogenous cavitation bubble depends primarily on the ultrasound peak negative pressure (PNP), but also on the abundance and solubility of gas in the tissue/material being sonicated. ‘Stable cavitation’ is the term typically assigned to the forced oscillations of bubbles that maintain their integrity under continued acoustic driving. When PNP is large enough, during expansion the gas bubble interior becomes so tenuous it can no longer resist the pressure of the surrounding medium, and it will violently collapse via ‘inertial cavitation’, producing local fluid jets and shock waves that can damage or disrupt nearby structures [3].

Much prior work examining cavitation and ablation therapy has sought to enhance treatment through cavitation [1, 4, 5]. Several groups have implemented feedback control to sustain cavitation activity and further enhance temperature rise or induce mechanical damage [6–8]. Recently, Lu et al. used a four-sector, dual frequency array for cavitation-enhanced ablation [9]. They compared a number of factors including single- vs. multi-focal sonication, single- vs. dual-frequency, and sonication duty factor on the formation of ablation lesions and cavitation level. When ablating at 1.6 MHz they measured a 6-fold increase in lesion volume when using multiple foci at twice the input power (applied in half the time) of the single-focus case.

Cavitation is ideally avoided in mild-hyperthermia therapies (40–45 °C) where the goal is for targeted cells to maintain viability for subsequent treatment (e.g., chemotherapy), or to evoke specific biological process such as apoptosis or anti-inflammation signaling [10–12]. Mechanical damage or thermal necrosis--both of which may result from spontaneous cavitation--would likely interfere with these processes. Additionally, a number of heat-sensitive drugs and drug-delivery vehicles that can be activated in situ with targeted heating are under investigation [13–17]. These require sustaining mild temperature rise within a tissue volume for several minutes, typically at low acoustic pressures that limit risk of mechanical effects [18]. Researchers have demonstrated increased metastatic burden in a mouse model of melanoma using high power acoustic pulses (1 MHz, PNP > 8 MPa, 50 msec duration) in conjunction with microbubbles that act as cavitation nuclei [19]. These bioeffects likely depend on cavitation dosage, since others have achieved cavitation-aided drug delivery without negative bioeffects using similar acoustic pulses in the absence of microbubbles [20].

A system capable of applying mild-hyperthermia with limited risk of cavitation is therefore desirable. We investigated whether this can be achieved by broadening the therapeutic beam by use of multiple foci. In 2013 Partanen et al. presented work with a clinical MR + HIFU system showing a multi-focal, volumetric mild hyperthermia treatment for several minutes [21]. They compared focus switching via electronic steering with sonicating all points simultaneously and demonstrated that multi-focal sonication reduces peak negative pressure and peak temperature, while still maintaining sufficient focal energy deposition for therapeutic temperatures.

In the present study, we compare the effect of single- and multi-focal continuous wave (CW) HIFU on heating while quantifying cavitation activity. Three focusing scenarios at six acoustic powers were examined in tissue-mimicking phantoms, and two scenarios were examined in pork. During sonications, cavitation activity was monitored with US imaging probes outside the MRI environment, and temperature was measured using MR thermometry. Broad beams produced from multiple foci reduced cavitation and spread heating over a wide spatial region suitable for mild hyperthermia applications.

## Methods

### Multi-focal sonication

**Fig. 1:**
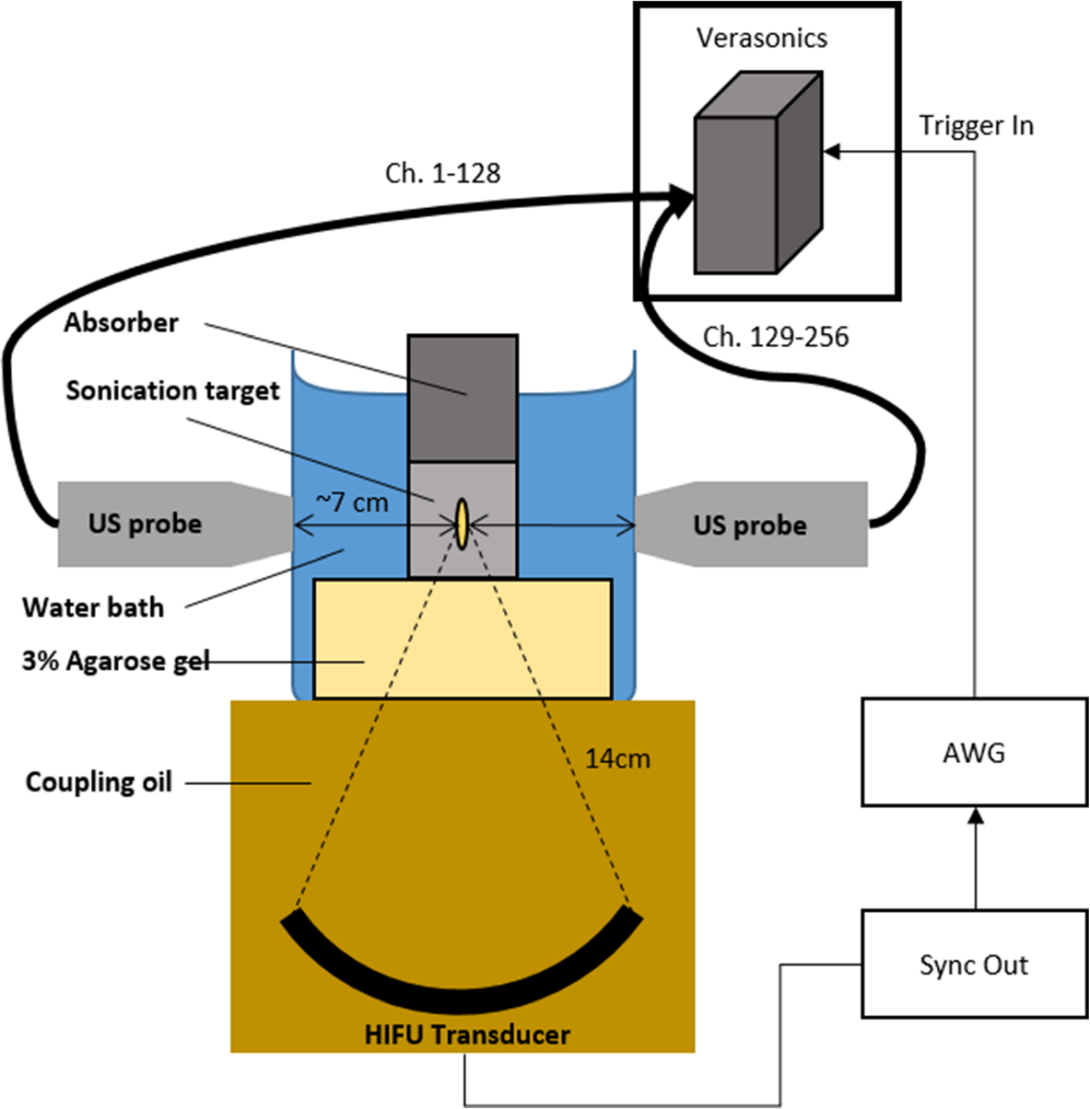
Experiment setup to monitor HIFU-induced cavitation

**Fig. 2:**
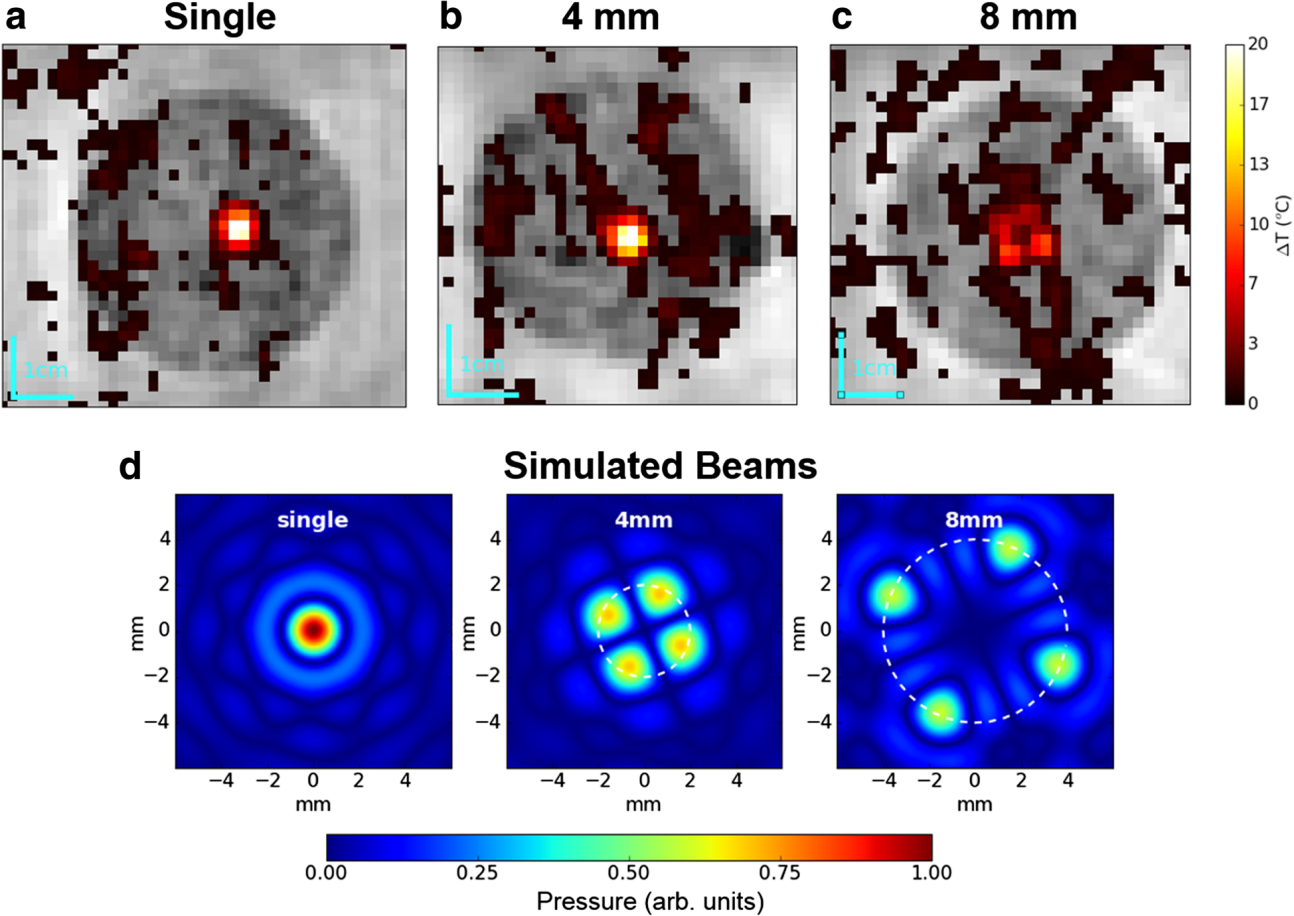
Depiction of the three HIFU beam geometries used in this study. **a**-**c** Example coronal MR images showing heat deposition from single focusing and the two multi-focus patterns. **a** Single focus beam. **b** Four points placed on an imaginary ring of diameter 4 mm. Individual foci were not resolvable with the given scan parameters, and thus appears similar to a single-focus in the image. **c** The same four points on a ring of diameter 8 mm. **d** Rayleigh-Sommerfeld simulated pressure field, normalized such that the single focus peak is unity, and the 4 and 8 mm foci are 0.7 and 0.6 respectively

We evaluated single and multi-focal sonication with the Philips Sonalleve MR + HIFU system. Sonications were performed with the Sonalleve V2 R3.2 system, a 256-element randomized sparse array transducer (element diameter 6.6 mm), spherically focused with a focal length of 14 cm and diameter approximately 14 cm at the opening face (Philips Medical Systems, Vantaa, Finland). Multi-focal beam patterns were generated in a manner similar to Partanen et al., which uses the method of Ebbini and Cain to compute array encodings [21, 22]. The Partanen study used an 8-mm ring pattern with 16 equally spaced foci in the transducer focal plane and sonicated for several minutes under temperature feedback control, based on the 8-mm treatment cell trajectory used by the Sonalleve in volumetric HIFU treatments [23]. Our multi-focal beam geometries were an 8-mm ring pattern and a 4-mm ring pattern, but in our case only four focal points were used, without feedback control. This pattern achieved more focused energy deposition than Partanen et al., resulting in heating within 30 s at low powers. The experimental setup is shown in Fig. 1, and multi-focal beams are shown in Fig. 2.

We then implemented array encodings on the Sonalleve transducer and sonicated tissue-mimicking gel phantoms and consumer pork chops, measuring temperature rise with MR thermometry. We implemented delays using the MATLAB programming interface MatHIFU, which provides a convenient interface for setting amplitudes and phases of the transducer elements, duration of sonications, sonication power, and scripted execution of commands that are outside the capabilities of the clinical HIFU interface [24]. The ‘RDCommand’ class was used to set the amplitudes, delays, and total power to the array. In single focus sonication, all element amplitudes were equal and phase offset set to 0°. In multi-focal sonications, amplitudes and phase offsets were determined according to Ebbini and Cain [22].

### Passive Acoustic Mapping (PAM)

To measure the spatial distribution of cavitation activity we used a common ultrasonic imaging technique. PAM closely resembles delay-and-sum image reconstruction, except that it uses one-way, receive-only delays. The equation used for image formation is given below.

1 $$ \mathrm{Image}(x) \propto {\displaystyle \int }{\left[{\displaystyle \sum_{i=1}^N}4\ \pi \left| x-{u}_{\boldsymbol{i}}\right|{v}_i\left( t + \frac{\left| x-{u}_{\boldsymbol{i}}\right|}{c}\right)\right]}^2 d t $$

Here, **x** is the reconstructed pixel location, **u**_**i**_ is the position and *v*_***i***_ the raw channel data of element *i.* The time argument in *v*_***i***_ represents the one-way delay from a coherent source at pixel **x** to element *i,* and the distance multiplier compensates for solid-angle spreading of a spherical wave*.* Time integration averages multiple image acquisitions together. This form of the reconstruction was referred to as “Time-Exposure Acoustics” (TEA-PAM) in Coviello et al. to delineate it from more advanced beamforming, but for simplicity we refer to it simply as PAM [25]. The equation for image pixel **x** is expressed as a proportionality because, although theoretically feasible, quantitative imaging would require a calibrated probe, accounting for the image point-spread function from measured data, and additional system transfer functions (digital filters, probe frequency response, etc.).

An open-top water tank with an agarose standoff held the sonication target such that its center was roughly at the HIFU geometric focus (14 cm). Acoustic windows were cut into opposite sides of the tank, covered and sealed with a thin plastic membrane to allow coupling of the US probes (Fig. 1). By using an imaging array PAM provided a broader spatial response over single-element cavitation monitoring due to the limited focal response of single-element transducers. We used two probes to ensure that “tail artifacts” inherent in PAM images that extend distally from the transducer face would not obscure cavitation sources in multi-focal sonications. Cavitation signals were acquired during continuous HIFU exposure using two ATL L7-4 128-channel ultrasound imaging probes connected to a 256-channel Verasonics imaging system. The Sonalleve HIFU generator provides a Sync Out for external triggering at the start of sonication, and this was used to trigger start of PAM acquisition. Due to the large amount of data generated by collecting cavitation images at a high frame rate, we limited HIFU sonications to 30 s, shorter than a typical hyperthermia treatment. After the initial trigger, acquisitions were made every 50 ms (20 per second) from both probes simultaneously. 2048 digital samples were recorded per acquisition, at a sampling rate of 20.8 MHz, resulting in 98.46 microseconds (2048/20.8) of data per each acquisition and an image reconstruction depth greater than 9 cm. Thus about 2 ms (20 * .09846 ms) of receive data was recorded for every second of CW HIFU, a receive duty factor of 0.2%. 600 frames were recorded in total, spanning a total of 30 s.

### Probe alignment and registration

The US imaging plane was approximately aligned to the HIFU focal plane in the following way. With the agarose standoff and a small amount of water, a low-power (1 W) continuous sonication was started, and the water level slowly increased. As the water surface height approached the focal plane, a slight fountain formed. Water was added and removed during this sonication via a siphon tube until the fountain intensity—indicated by the height of ejected water—was roughly maximized. This qualitative procedure was sufficient since the full-width half-max (FWHM) of the focus in the axial direction is long (~1 cm). The two imaging probes were then attached to optical table rails and slid towards the coupling window. The center line of each probe face was aligned with the tank water level, coupled, and secured against further motion. The remainder of the tank was then filled with water. On completion of this procedure, the HIFU focal plane was approximately centered within the elevational focus of the L7-4 transducer (FWHM of 6 mm at the optimal focusing depth).

Image registration was required to construct PAM images from received echoes on both probes. To compute the registration, several fiducial measurements were conducted. First, the two probes were operated in pulse-echo mode while a point source (steel rod with diameter of 2 mm) was positioned at several locations in the image field. Second, the therapeutic transducer at high power (60 W acoustic) briefly sonicated (5 s) an agarose phantom with the beam steered to several positions, which served as cavitation point sources in the resultant PAM images. These calibration methods provided two consistent sets of common fiducial points in the image space of probes 1 and 2. Probe 2 was then registered to the probe 1 space using a rigid transform. Jointly reconstructed images are reported relative to probe 1. Registration yielded a probe separation of roughly 13.6 cm, which agreed with the measured tank width.

### MR temperature mapping

Thermal maps were generated with a 3T Philips Achieva using the proton-resonance frequency shift method (PRFS), where the phase change between two acquisitions is proportional to temperature change [26]. The sequence was a multi-slice gradient echo planar imaging sequence (FEEPI with TE = 16 ms, TR = 24 ms, EPI factor 11, voxel size 1.8 × 1.8 mm and 3 mm slice thickness, with field-of-view (FOV) of 222 × 170 × 18 mm). Coronal slices were used to verify multi-focal heating, and sagittal slices aligned with the HIFU focus in the center slice were used for thermal measurements. A 3D stack of images was acquired with a dynamic scan time of approximately 1.3 s per volume. Temperature distributions were examined in a region-of-interest (ROI) with approximate dimensions of 16 × 12 × 12 mm, with the long dimension along the HIFU direction. ROI size was chosen to fully encapsulate the focal zone and allow a small margin to account for heat diffusion. Since the PRFS method uses phase difference between each dynamic and a baseline scan, a large temperature change will eventually cause computed phase differences to roll-over from π to –π, falsely indicating a large negative temperature swing. This rollover was corrected by locating voxels with phase difference less than -π/2 (with respect to baseline) and adding 2π. Phase-drift was not corrected due to the short duration of scans (40 s). For visualization, thermal maps are overlaid on grayscale magnitude images.

### Sample preparation

Tissue-mimicking gel phantom was prepared by mixing agar powder (Now Foods, Bloomington, IL) and 400 grit silicon carbide powder (Beta Diamond Products, Anaheim, CA) into de-gassed water. Agar concentration was 3% and graphite concentration was 1% (weight [g]/volume percent [ml]). The liquid solution was heated to 90 °C then allowed to cool for several minutes while stirring, then poured into a 5-cm diameter glass cylindrical phantom mold placed in a cool water bath to decrease cooling time. After hardening, the cylinder gel was removed and cut lengthwise into sections approximately 3.5 cm long. This allowed creation of a large batch of phantoms at once. Pork chops were purchased from a local grocery and samples prepared by removing fatty tissue pieces and cutting into cubic sections approximately 3 cm thick.

### Phantom and pork sonications

A total of 72 phantom sonications and 24 pork sonications were performed. Half of the sonications were performed with US imaging to assess cavitation and half within the MRI system to measure temperature. Phantoms or tissue samples were always replaced between sonications to ensure that heating or depletion of cavitation nuclei did not affect subsequent measurements. Cavitation imaging trials were first performed with the transducer bed outside of the magnet room. Using the MatHIFU interface, applied power was 5, 10, 20, 40, 60, or 80 acoustic watts for single focus and two multi-focal patterns (diameters of 4 and 8 mm). Temperature mapping experiments were done within the magnet: the US imaging probes were removed, the bed was rolled into the magnet, and the same protocol repeated. Cavitation and MR imaging of phantoms was repeated once, with fresh phantoms. Pork sonications followed the same procedure comparing single and 8-mm multi-focal geometry.

Lesion formation was observed after single-focus sonications in the pork samples at 40 W and above. No lesions were observed in the multi-focal case. Since solid lesions accelerate heating, and likely change the probability of cavitation, 60 W and 80 W single-focus sonications of pork were not repeated in the MR scanner. PAM image data were collected for these cases.

### Data analysis

US and MR data were analyzed offline using custom Python scripts. Experimental data was grouped according to sonication power level, focus geometry, and target material. PAM images were reconstructed using the beamformer in equation (1) with pixel size of 0.2 × 0.2 mm. The magnitude in reconstructed PAM images is proportional to cavitation source strength. US probe voltages are encoded as signed 16-bit integers by the Verasonics acquisition hardware. All numerical values presented from ultrasound channel data are in arbitrary units and were acquired with consistent gain settings that avoided saturation.

To measure cavitation source strength we computed the time-domain signal variance and broadband (non-harmonic) spectral noise observed in each acquisition. Variance was computed per channel using the raw channel data from each probe. The summed variance over all 256 channels was then used as a measure for cavitation (referred to as “Ch. Variance” in figure labels). Broadband noise was computed by first taking the Fast Fourier Transform of each channel. Harmonics and ultra-harmonics were removed with a notch filter (-3 dB passband width of 0.4 MHz) centered at multiples of 1, 1.5, 2, 2.5, etc. of the 1.2 MHz HIFU frequency. Broadband noise was calculated as the sum over the filtered spectrum. Both broadband noise and total variance were compared to a PAM-derived measure of cavitation, which we expressed as the mean pixel value of a reconstructed PAM image (i.e., the average of a reconstructed image).

## Results

### Multi-focal sonications generated distinct foci

The single focus and two multi-focal sonications generated expected heating patterns after a 10-s sonication at 40 W in phantoms (Fig. 2). Simulations suggest the multi-focal sonications are roughly 70% (4-mm) and 60% (8-mm) of the single focus peak negative pressure (PNP), and these were verified at low power (1 W) with a needle hydrophone. During the 4-mm sonication, four foci were not individually resolved most likely due to the scan having spatial resolution comparable to the distance between adjacent foci, although the hot spot produced was broader than a single focus. The 8-mm sonication produced individual foci that could be visualized with MR thermometry.

### PAM imaged simultaneous cavitation nuclei

**Fig. 3:**
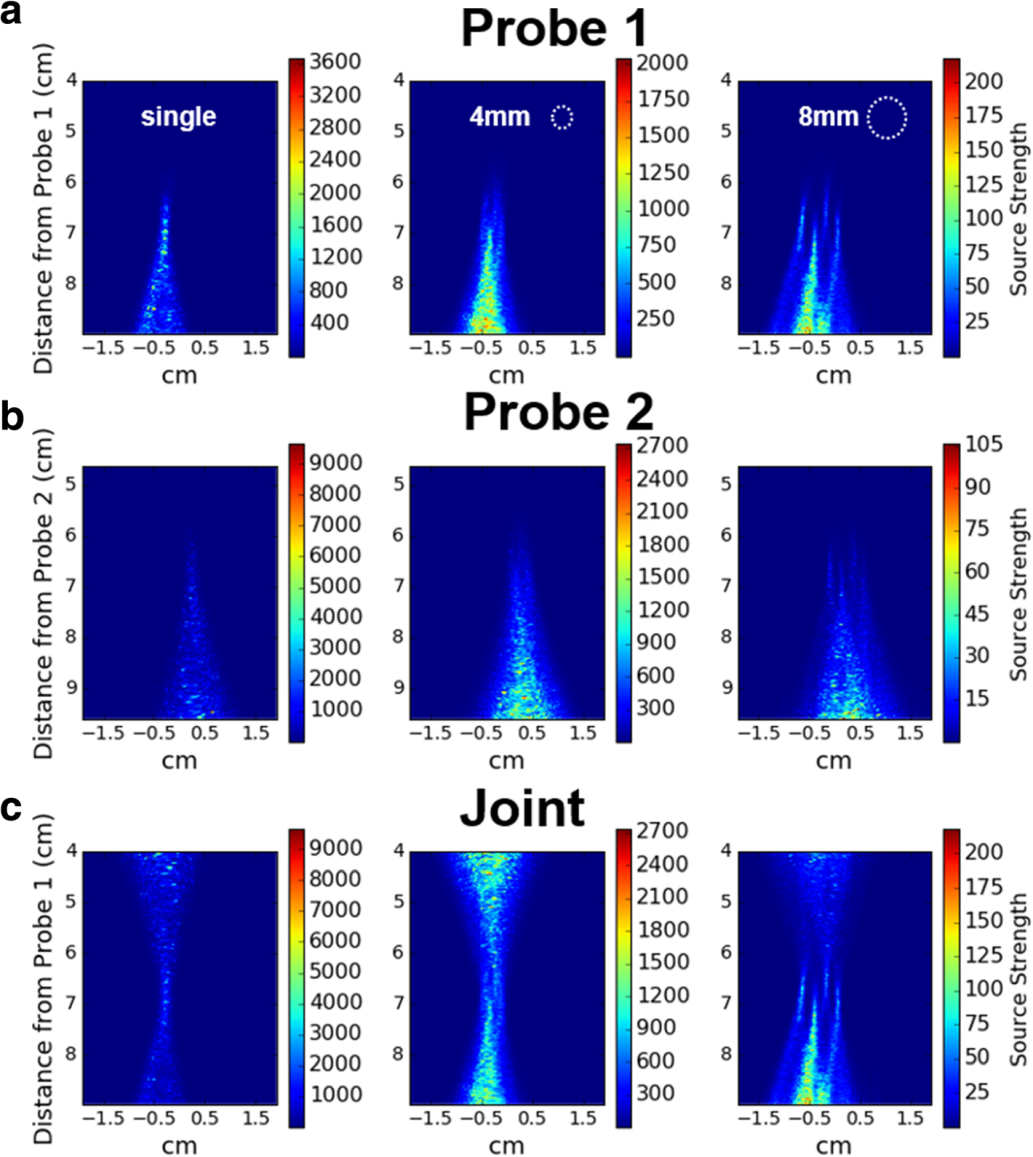
Example PAM images for a set of 60 W sonications in phantoms, time-integrated over all 30 s of HIFU + PAM acquisition. The columns are as follows--Left: single-focus. Middle: 4-mm multi-focus. Right: 8-mm multi-focus. Rows (**a**) and (**b**) show Probe 1 and Probe 2 reconstructed according to Eq. (1). In row (**c**) Probe 2 was registered to Probe 1 and both probes reconstructed in the co-registered pixel domain. In PAM images tail artifacts can be easily recognized. The ratio of apparent tail strength to magnitude of the foci is larger with multi-focus. It can also be seen that, in each multi-focus case, the focal point furthest from the probe has a larger tail effect. This is perhaps due to reverberation and backscattering of waves from cavitation at the closer foci

The PAM technique was capable of resolving foci in single and multi-focal sonications in the phantom. Individual foci can be clearly resolved in the 8-mm multi-focal case but are less distinct in the 4-mm case (Fig. 3). Tail artifacts appeared in every PAM image, but when comparing the near-field of one probe to the far-field of the other, no additional cavitation sources were present other than the HIFU foci. In multi-focal sonications the farthest focal point from the perspective of each probe had the brightest tail artifact in all cases.

### PAM correlated with standard cavitation metrics

**Fig. 4:**
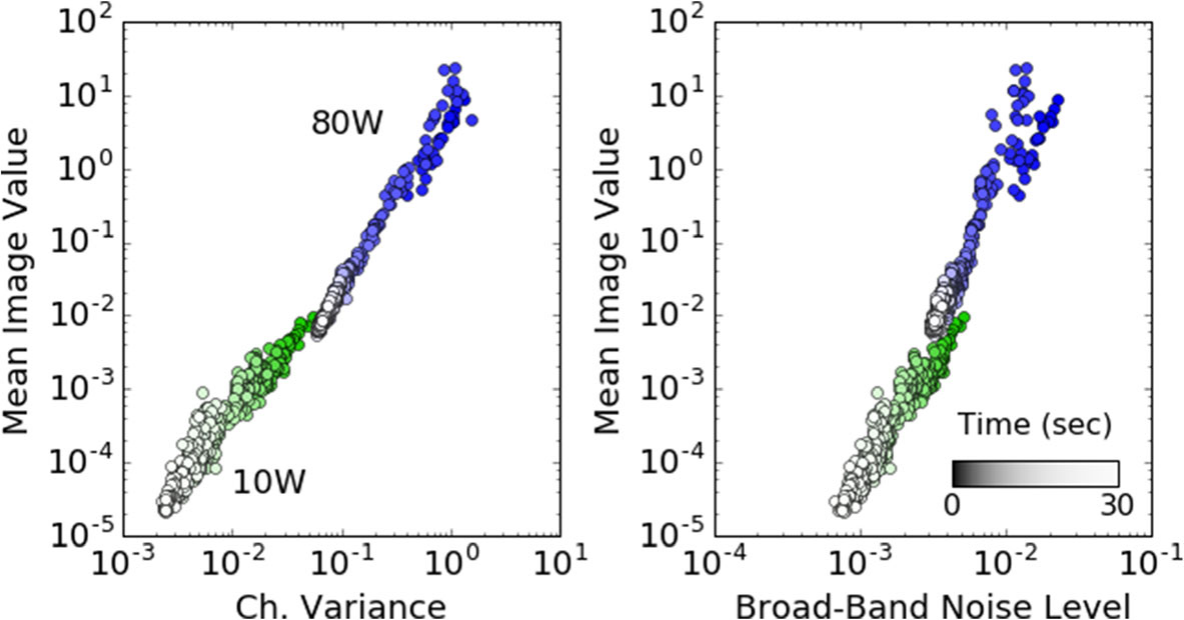
Example of PAM image magnitude (mean of pixel values) vs. commonly reported measures of cavitation activity (arbitrary units) for a 10 W and 80 W, 30-second phantom sonication. The tight correlation suggests PAM image magnitude is equivalent to variance and broadband noise as a proxy for cavitation activity. Each point is computed from a single ultrasound acquisition, and the full set of 600 acquisitions is shown for 10 W and 80 W. Channel variance is the sum of raw channel variances (proportional to receive volts-squared). Broad-band noise level is the total amplitude of the frequency spectrum (proportional to volts), once harmonic and ultra-harmonics have been filtered out. In both plots the x-axis values were computed per channel and then summed. The color saturation gradient represents sonication time, as is shown in the color bar on the right. The most saturated color is at time 0, and the least saturated is at 30 s. Quantities remained correlated even after bubble depletion in the first several seconds

Passive US monitoring produced results consistent with expectation. First, we observed a nearly one-to-one relationship between mean PAM value and channel variance, and between PAM value and broadband noise. The mean PAM image magnitude correlated strongly to the time series variance and to the square of the broadband noise level (Pearson’s r > 0.9 in all cases). Figure 4 illustrates this correlation for 10 W and 80 W single-focus sonications in phantoms.

### Multi-focal sonication reduced cavitation

**Fig. 5:**
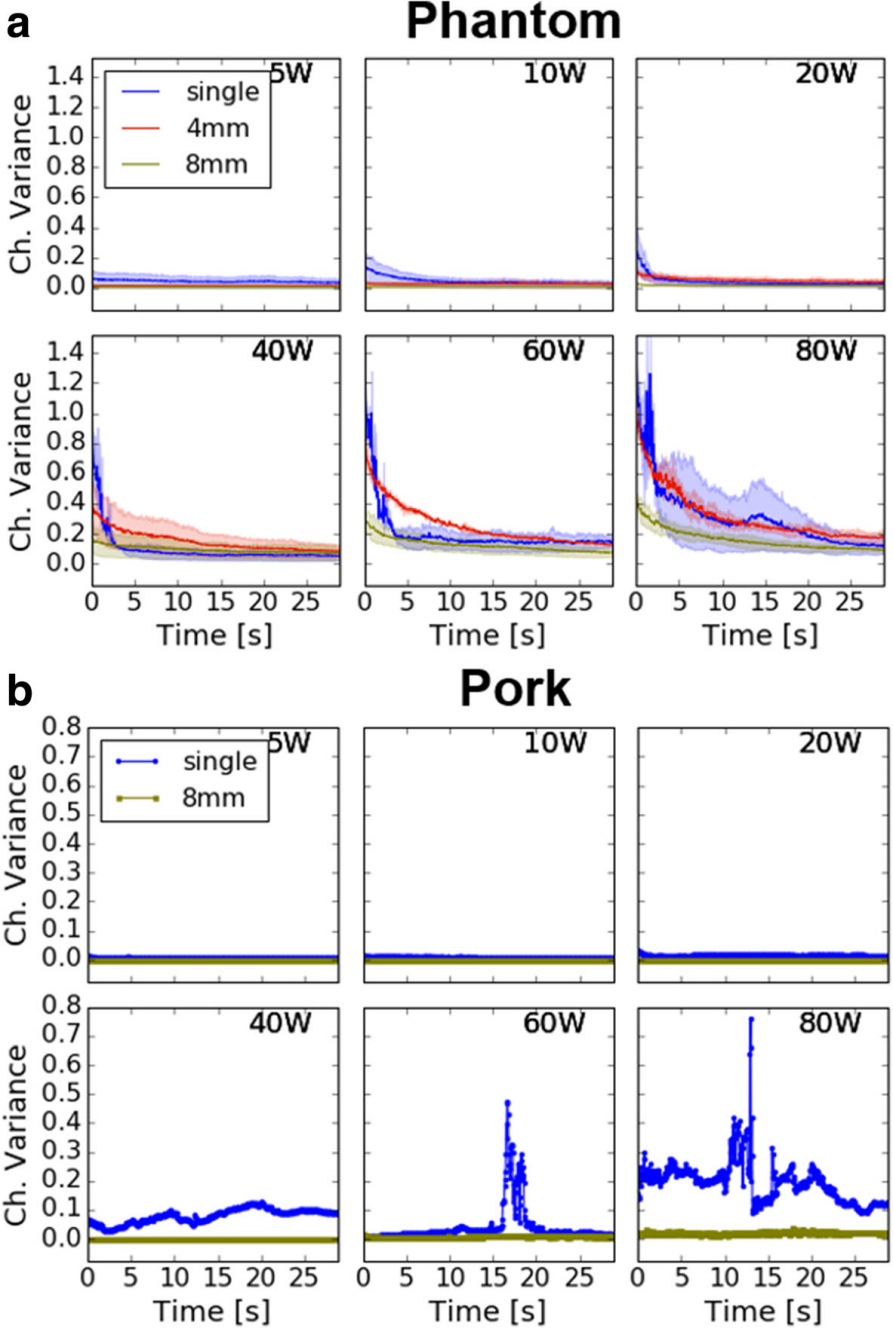
Time history of total channel variance as a measure of cavitation activity for 30 s of HIFU. Each point represents one acquisition and is the sum of the 256 raw channel variances. **a** Averaged value from phantom trials, as well as the min and max values observed. The observed exponential decay is consistent with cavitation depletion and transient, noisy signals are indicative of cavitation bubble collapse. **b** Result of pork trials. In pork, a thermal lesion was observed after the 40 W single focus sonication

**Fig. 6:**
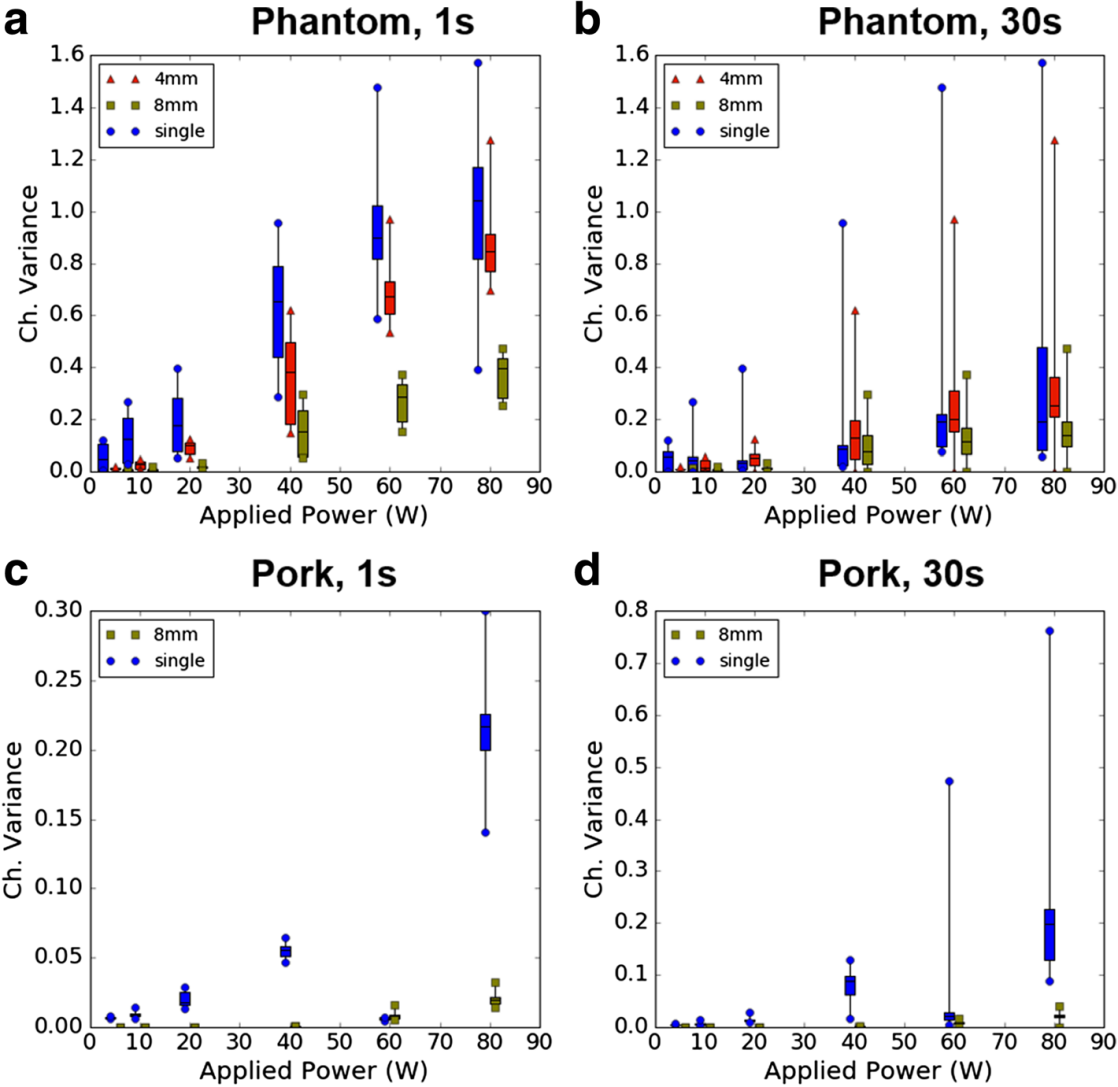
Distribution of measured cavitation activity (summed channel variance), vs. total power applied to the array for one set of sonications at 5, 10, 20, 40, 60, 80 W. *Box* locations are offset along the x-axis to allow side-by-side comparison. Each *box* represents the distribution (min-max, and 25, 50, 75th percentiles) of cavitation activity. **a** and **c**, distribution within the first second. **b** and **d**, distribution over the full 30-seconds

At all powers examined, cavitation activity (as measured by channel variance) had an initial high amplitude onset followed by an exponential decay towards zero as sonication progressed. In phantoms, a majority of cavitation occurred within the first 4 s of sonication with the longest decay times occurring in multi-focal cases (Fig. 5). Cavitation in pork was less consistent, with the 40 W case giving a consistent low amplitude channel variance while the 60 W and 80 W cases showed high amplitude peaks with fast decay multiple seconds into the sonication. Within the first second, the mean channel variance during single focus sonication was higher than multi-focal in both the phantom and pork preparations (Fig. 6a, c). Though cavitation activity was always initially highest using the single-focus beam, the 4-mm multi-focal beam had similar average variance to the single focus when measuring cavitation over the entire 30 s sonication, most likely due to a rapid depletion of cavitation bubbles at the single focus. We observed reduced cavitation in the 8-mm case at all powers.

### Multi-focal sonications reduce peak heat

**Fig. 7:**
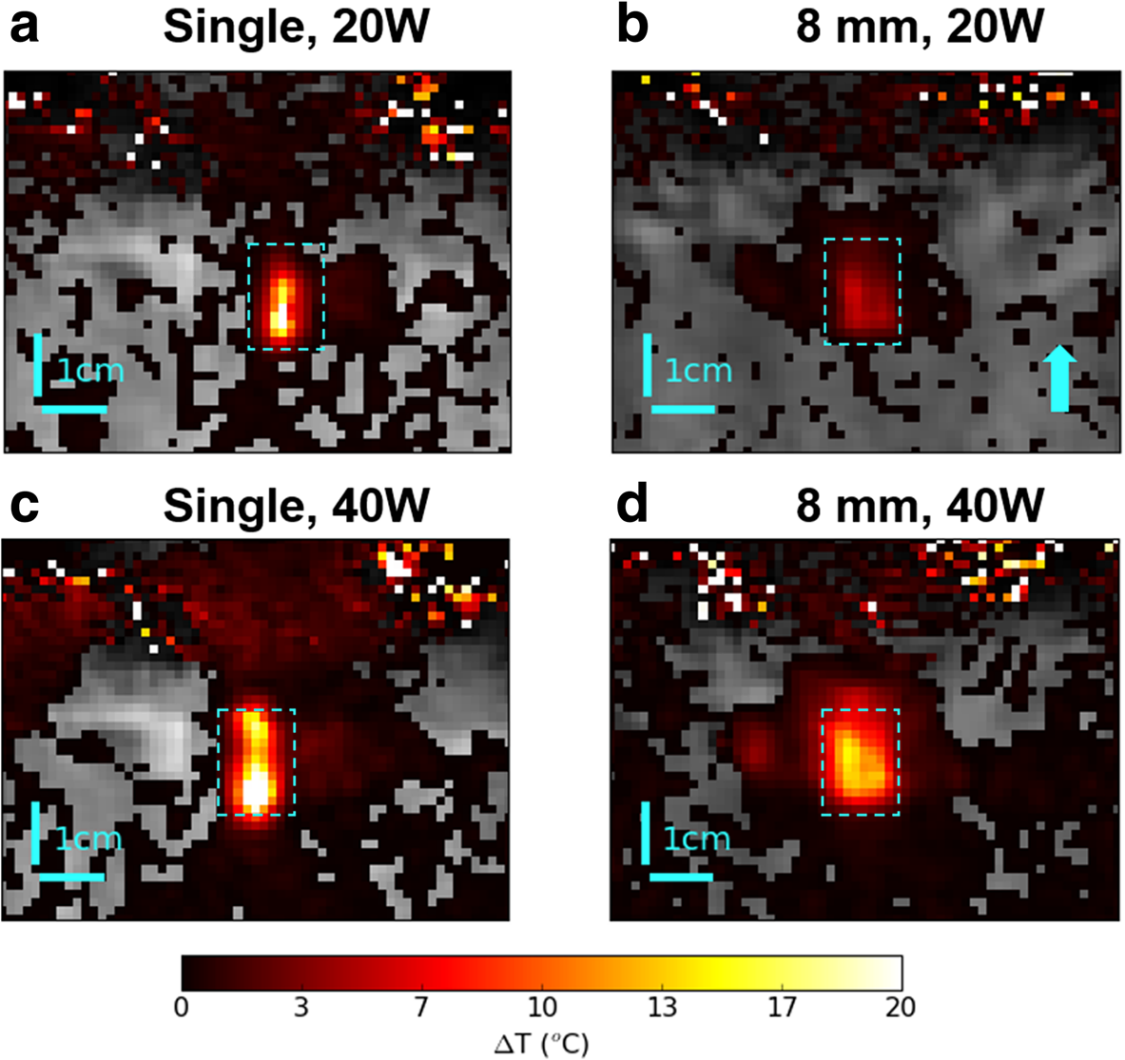
Zoomed view of the center slice (sagittal) of MR mapping volumes for 20 W and 40 W sonications in pork after 30 s of HIFU. The *arrow* indicates direction of HIFU propagation. The *dotted line* represents ROI cross-section. Note: after sonicating at 40 W a surface lesion was observed at the pork interface (bottom of the ROI in (**c**)). Note: the color scale has been windowed to 0–20 °C to emphasize contrast in multi-focal sonications

**Fig. 8:**
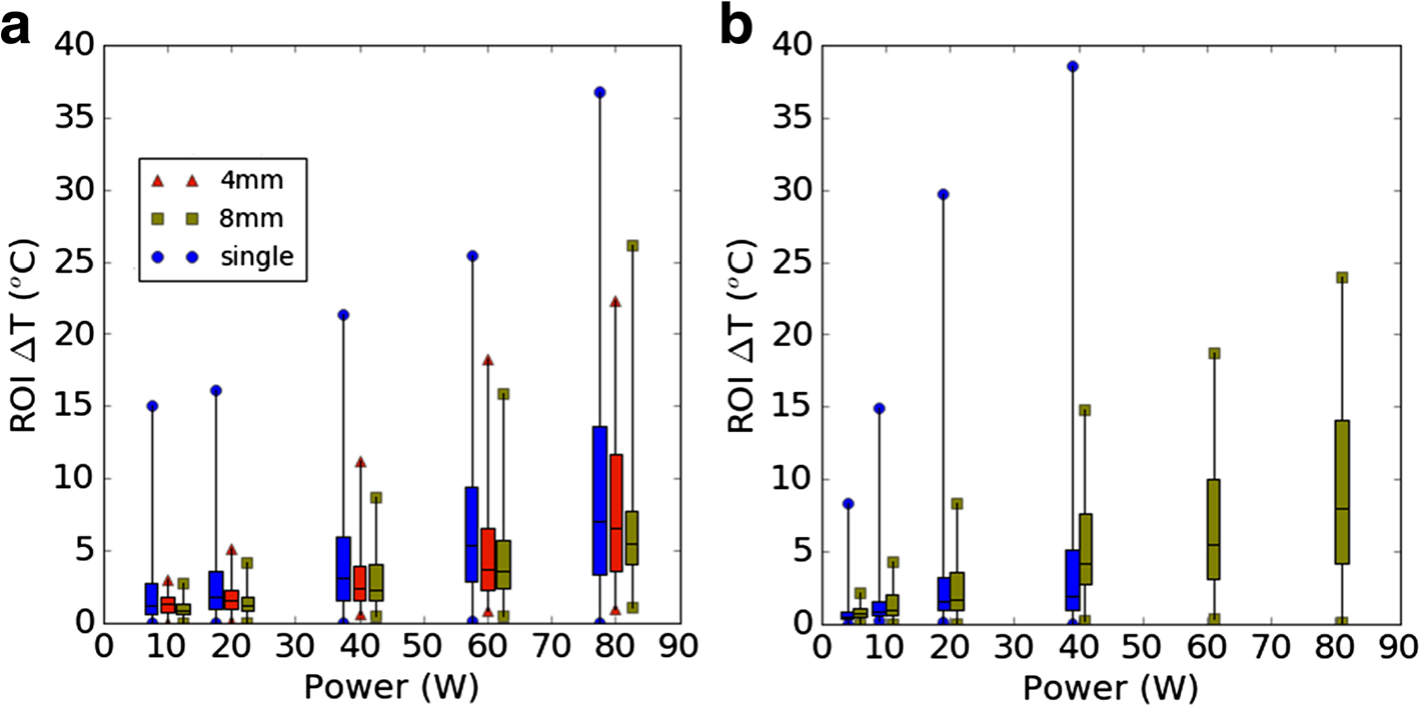
Distribution of voxel temperatures in the focus ROI in (**a**) phantom, and (**b**) pork after a 30 s sonication. Min/max and percentiles levels corresponding to 25, 50, 75% of voxels are plotted. Multi-focusing has lower peak temperature more voxels in the mild hyperthermia range. 5 W sonication data is omitted from (**a**) to prevent crowding. 60 W and 80 W single-focus sonications were not repeated in the scanner with pork due to formation of thermal lesions at 40 W

**Fig. 9:**
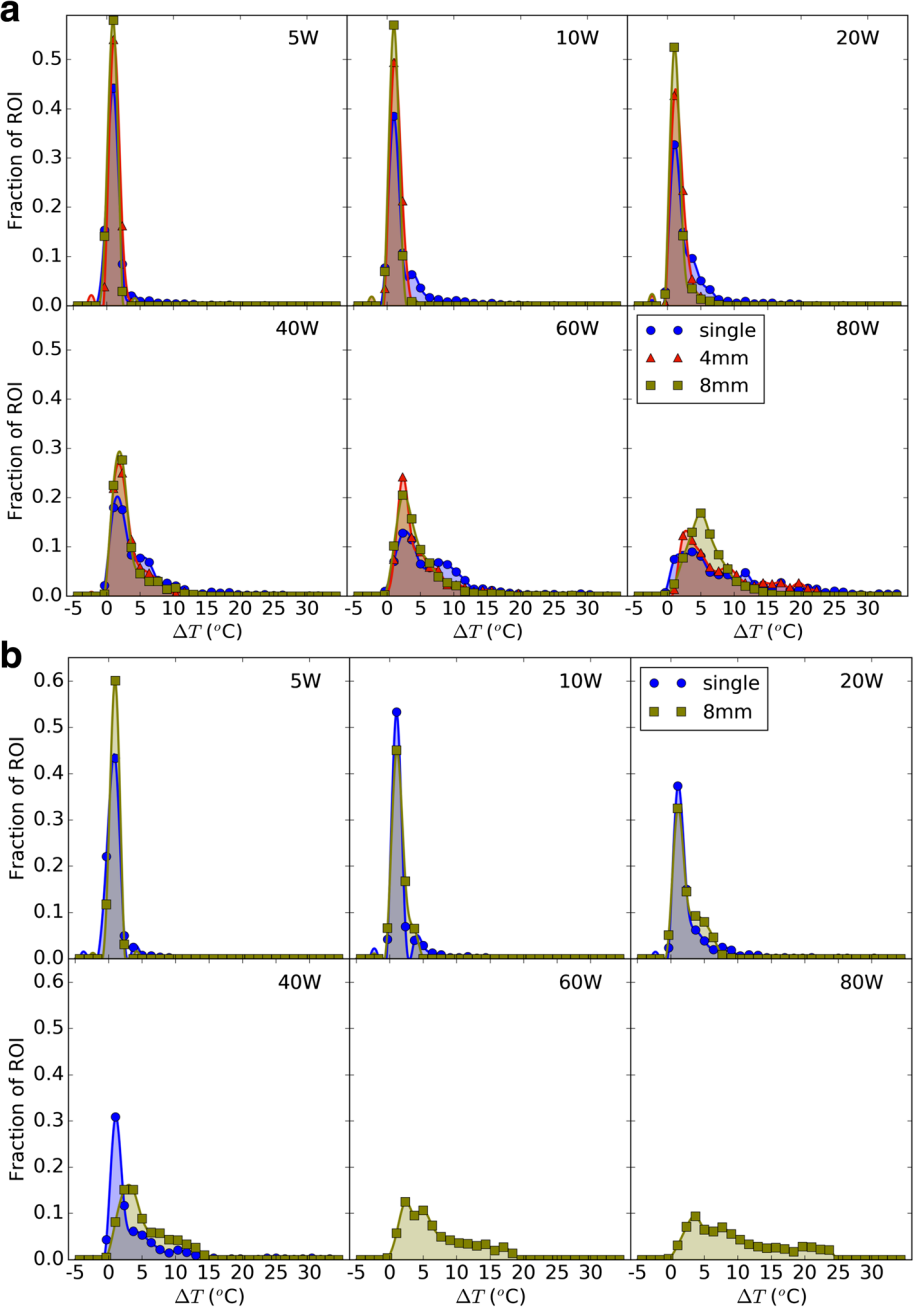
Distribution (histogram) of voxel temperatures in the focus region-of-interest (ROI) in (**a**) phantom, and (**b**) pork, after 30 s of constant-power, continuous wave HIFU

**Fig. 10:**
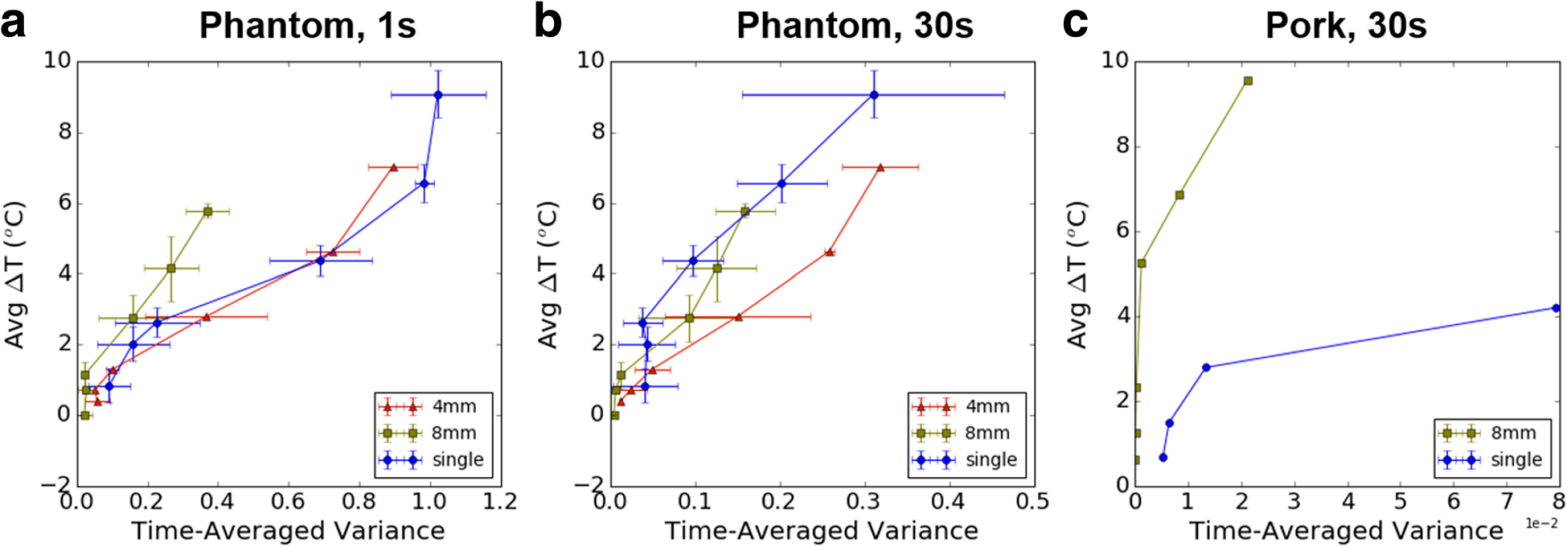
Final temperature rise after 30 s of HIFU (averaged over the ROI) vs. cavitation activity. Points correspond to 5, 10, 20, 40, 60, and 80 acoustic watts (in pork, single-focus MR data wasn’t collected above 40 W due to lesion formation). **a** Averaging over 1-s in phantoms (prior to bubble depletion) shows less cavitation at a given input power in both 4 mm and 8 mm cases. **b** Averaging over the full 30 s sonication, 8 mm multi-focusing achieves an average rise in the mild heating range (+3-8 °C) above 20 W with generally less cavitation at each input power level. At 40 W total cavitation activity is comparable between the two, but this is likely due to initial rapid depletion of bubbles in single focus (see Discussion). **c** Negligible cavitation activity was observed with multi-focusing in pork. The large jump in channel variance at 40 W, pork, single-focus may be a result of lesion formation, cavitation, or both

In all cases examined, single-focusing resulted in higher peak temperature and more focused heating at a given input power. At 20 W and 40 W the single focus heating was 21 °C and 24 °C greater than the 8-mm multi-focal case, respectively, and the temperature distribution across the ROI was more uniform in the multi-focal case (Fig. 7). Figure 8 displays the distributions as a box-and-whisker plot for each focusing case, at 10, 20, 40, 60 and 80 W acoustic power. Figure 9 displays the same data as a normalized histogram, from which the fraction of the ROI at a given temperature can be seen. A larger max-min spread of temperature values was observed with single-focused beams, while multi-focusing resulted in narrower voxel distributions with more voxels clustered in the mild hyperthermia range. For example, at 40 W in pork, the peak temperature rise was +39 °C in single-focus and +15 °C in multi-focal, but the median was +2 °C vs +4.5 °C, respectively. Broadening the beam made heating more diffuse and reduced total cavitation (Fig. 10).

## Discussion

### Summary of results

In this study, we have demonstrated that by broadening the therapeutic HIFU beam with multiple foci, a large reduction in cavitation activity can be achieved while still maintaining temperature increases in the range of mild hyperthermia. Similar multi-focal approaches to reduce peak pressure have been previously demonstrated to be effective for mild hyperthermia, and our study is the first to measure cavitation activity during multi-focal heating [21]. We used diagnostic imaging arrays to receive cavitation signals over the broad multi-focal area, and implemented PAM to image the spatial location of cavitation foci in phantoms. Standard metrics of cavitation (broadband noise level and channel variance) linearly correlated to the mean PAM image values, and multi-focal sonications generated less cavitation activity and more diffuse heating. These characteristics make the proposed sonication method ideal for therapeutic applications where heating in the absence of cavitation is desired.

### PAM imaging of multi-focal cavitation

We spatially mapped simultaneous cavitation foci during multi-focal sonication using PAM to generate images with cavitation foci geometrically arranged in the expected focal pattern. The US probes demonstrated the probe tail artifact distal to the transducer often reported in PAM literature (Fig. 3). Multi-focal cases in pork did not result in clear images corresponding to the HIFU foci (data not shown), and single-focus images in pork were blurred compared to corresponding images of phantoms. PAM magnitude was also significantly lower when sonicating pork. Although standard PAM has been performed in the presence of microbubbles in heterogeneous media [27], we did not use any exogenous agents to act as cavitation nuclei. Our experiments instead relied on spontaneous cavitation of endogenous gas bodies, which generally provide much lower cavitation SNR than microbubbles. PAM has not been used (to our knowledge) to detect endogenous cavitation in heterogeneous media. We hypothesize that lower SNR, increased attenuation and tissue interfaces hindered the algorithm’s performance in pork, and that PAM reconstruction could potentially be improved using more robust beamforming, such as the minimum covariance method used by Coviello [25]. However, multi-focal sonications in pork were evident from MR thermal imaging, despite the lack of clear PAM reconstruction.

### Temporal evolution of cavitation

One goal of this study was to examine the temporal evolution of cavitation activity during different sonication geometries. In phantoms, the channel variance was predictable with cavitation activity occurring at the onset of sonication. Cavitation was highest during single focus sonication and depleted rapidly, while multi-focal sonication resulted in cavitation that was initially lower and decayed more slowly. The exponential decay seen in Fig. 5a is consistent with a gradual depletion of cavitation bubbles through inertial cavitation, similar to observations that have been previously reported [7, 28]. In Fig. 5a, transient bursts on top of exponential decay can be seen in the single-focus channel variance curves, but not to the same extent in the multi-focal curves. Similar transient signals have been reported in the past, and are likely caused by the spontaneous nucleation and subsequent collapse of a new endogenous bubble, or by movement of a bubble into/out of the probe focus [7, 29].

During the first second of ultrasound acquisition from phantoms (Fig. 6a), the median and max channel variance in the 4-mm and 8-mm cases were below single-focus values at every power level. However, when considering the entire 30-s sonication, the difference between single- and multi-focal channel variance decreased substantially, with the 4-mm case having slightly greater median and average channel variance compared to the single focus above 40 W (Fig. 6b). We hypothesize that cavitation nuclei depleted rapidly in single-focus sonications, resulting in similar channel variance to the 4-mm case when averaged over 30 s. In the broadest beam (8-mm) average cavitation was lower at all powers (Fig. 10b).

In pork samples, multi-focusing produced lower channel variance than single-focusing at all power levels. Channel variance remained at a low level in sonications below 40 W. At 60 W and 80 W, short-lived spikes were present, potentially signaling spontaneous nucleation and collapse of cavitation bubbles at the focus. Solid lesions appeared at 40 W and above in pork. The presence of both lesion and sporadic acoustic bursts is consistent with a previously reported association between lesion formation and the acoustic signature of cavitation [30]. There were no acoustic spikes observed in multi-focal sonications of pork samples, nor were visible lesions formed.

### Diffuse heating with multi-focal ultrasound

In phantoms and pork, both multi-focal patterns had lower peak temperature and generated mild heating (∆T = 3–8 °C) more consistently spread over a volume of interest than the single focus. In phantoms, most cavitation was observed soon after the sonication and prior to significant heating. This suggests the reduction in cavitation activity was primarily due to smaller acoustic pressures when using multiple foci. Therefore, these data confirm the hypothesis that, in mild hyperthermia applications where cavitation is undesirable, multi-focusing is a potential option to achieve the desired temperature elevation at reduced pressure.

### Study limitations

It should be noted that the sonication protocols used do not reflect a practical hyperthermia treatment protocol. For example, voxel temperatures were allowed to exceed the mild range since feedback control was not used. Our group has experience with mild hyperthermia feedback (e.g., Poorman et al. [31]) but the present study was instead designed to observe the onset of cavitation at matched powers, and we showed that it is less likely to occur in multi-focusing at a given input power.

Temperature rise and cavitation threshold were dramatically different between phantoms and pork. In our study, mild-hyperthermic heating was achieved in pork below the cavitation threshold. Pork samples had a higher thermal absorption and higher cavitation threshold than phantoms. Thus the multi-focal approach may be most advantageous when sonicating tissues with low cavitation threshold, such as adipose tissues [29, 32].

We also traded temporal resolution for spatial coverage by using a low receive duty factor (0.2%) in our study, similar to what has been used previously in cavitation monitoring [28]. With this duty factor, PAM yielded ~1 MB per image frame (600 MB per sonication). Echoes from repeated cavitation events (<50 ms) could be missed in our acquisitions, though results from other studies of transient cavitation with better temporal resolution suggest many cavitation bursts are longer than 50 ms when applying CW or long HIFU pulses [6, 7]. A systematic study of cavitation burst duration in continuous wave HIFU with 100% receive coverage was not found in the literature, however the work of Li et al. is informative. That study used a series of 60, 1 ms HIFU pulses to measure the threshold, probability, and persistence of cavitation in different materials. They found a rapid transition to 100% cavitation probability above the cavitation threshold of 3 MPa in agarose [32]. Since we were likely above the cavitation threshold in most phantom experiments (based on exponential decay of variance), this suggests that even at the 20 Hz frame rate cavitation events could be detected. In pork it is less clear what events may have been missed, since there are clearly short transients that were detected lasting just 1–2 acquisitions at 40 W and above (small ‘blips’ in Fig. 5b).

Finally, our study would benefit from robust quantification and mapping of the acoustic field produced by the Sonalleve at all power levels tested, since at higher powers, increasing acoustic non-linearity would change the ratio of PNP. Ideally, an optical hydrophone would be used to measure peak negative pressure in each sonication scenario, but this was not available. Recasting the results from acoustic watts to free-field acoustic pressures at the foci would help define PNP thresholds for cavitation.

### Potential applications

The general conclusion from this study is that diffusing the beam of a spherically-focused HIFU transducer may be advantageous when performing mild-hyperthermia, because the risk of cavitation can be reduced. A simple way to achieve beam diffusion is by using multi-focal sonications with an array transducer or alternatively, single-element transducers could be diffused via an acoustic lens [33]. Increasing the number and spacing of foci diffuses the beam and decreases peak negative pressures. We have demonstrated this trend in two specific patterns. However, if the beam becomes too diffuse then it may be difficult to achieve therapeutic temperatures or to target specific tissues. Also there is a risk of axial grating lobes reaching therapeutic intensities, so simulations and hydrophone measurement should be used to understand beam shape relative to the therapeutic application. In all cases, beam diffusion can reduce cavitation, but depends on the specific transducer (curvature, aperture, focal gain, sonication frequency, etc.), as well as the morphology of the targeted tissue. Other possible advantages of multi-focal sonication such as shaping of the heated region are discussed in Partanen et al. [21].

The simultaneous broadening of the heated zone and reduction in cavitation level may be advantageous in a number of ultrasonic therapies where heating in the absence of cavitation is desirable. Studies using mild hyperthermia to activate biological pathways such as apoptosis or immune responses might use this technique to avoid confounding effects from mechanically-induced cell damage, which has been shown to directly correlate with inertial cavitation dose [34]. Many researchers are currently studying temperature-sensitive drug carriers loaded with chemotherapeutics. Thermally sensitive liposomal carriers loaded with doxorubicin have been used pre-clinically with ultrasound-induced hyperthermia to effectively treat several cancer models [13–17]. Molecular dynamic simulations have suggested that protein structures can be significantly modified by acoustic shockwaves and nanometer scale jets, and cavitation-produced free radicals may also alter the efficacy and toxicity of therapeutic molecules [35, 36]. The multi-focal methods used in our study can deliver mild hyperthermia in the absence of cavitation and may be preferable in instances where mechanical cavitation could hinder dose delivery.

## Conclusion

Multi-focal HIFU was used to achieve mild temperature elevation with reduced cavitation activity. Cavitation activity was monitored using the variance and PAM on two transducers. PAM was able to resolve multiple foci in the phantoms and image magnitude was highly correlated with channel variance in both pork and phantoms. Multi-focal HIFU can be used in the future in conjunction with feedback control to perform mild hyperthermia treatments with less probability of cavitation.

## Abbreviations

US: Ultrasound
PAM: Passive acoustic mapping
ROI: Region-of-interest
